# Deep neck infections with and without mediastinal involvement: treatment and outcome in 218 patients

**DOI:** 10.1007/s00405-021-06945-9

**Published:** 2021-06-23

**Authors:** Thomas Gehrke, Agmal Scherzad, Rudolf Hagen, Stephan Hackenberg

**Affiliations:** Department of Otorhinolaryngology, Head and Neck Surgery, University Hospital Würzburg, Josef-Schneider-Str. 11, 97080 Würzburg, Germany

**Keywords:** Deep neck infection, Mediastinitis, Surgical drainage, Microbiology, Tracheotomy

## Abstract

**Purpose:**

Infections of the deep neck, although becoming scarcer due to the widespread use of antibiotics, still represent a dangerous and possibly deadly disease, especially when descending into the mediastinum. Due to the different specialities involved in the treatment and the heterogenous presentation of the disease, therapeutic standard is still controversial. This study analyzes treatment and outcome in these patients based on a large retrospective review and proposes a therapeutic algorithm.

**Methods:**

The cases of 218 adult patients treated with deep neck abscesses over a 10-year period at a tertiary university hospital were analyzed retrospectively. Clinical, radiological, microbiological and laboratory findings were compared between patients with and without mediastinal involvement.

**Results:**

Forty-five patients (20.64%) presented with abscess formation descending into the mediastinum. Those patients had significantly (all items *p* < 0.0001) higher rates of surgical interventions (4.27 vs. 1.11) and tracheotomies (82% vs. 3.4%), higher markers of inflammation (CRP 26.09 vs. 10.41 mg/dl), required more CT-scans (3.58 vs. 0.85), longer hospitalization (39.78 vs 9.79 days) and more frequently needed a change in antibiotic therapy (44.44% vs. 6.40%). Multi-resistant pathogens were found in 6.67% vs. 1.16%. Overall mortality rate was low with 1.83%.

**Conclusion:**

Despite of the high percentage of mediastinal involvement in the present patient collective, the proposed therapeutic algorithm resulted in a low mortality rate. Frequent CT-scans, regular planned surgical revisions with local drainage and lavage, as well as an early tracheotomy seem to be most beneficial regarding the outcome.

## Background

Bacterial infections and abscesses are frequently occurring diseases in the head and neck. Due to the connection of the cervical fascial spaces, however, initially localized infections can spread into the deep neck rapidly and even descend into the mediastinum [1]. Most of these descending infections have pharyngeal (36–47%) or odontogenic (33–45%) origins, from where they descend along the parapharyngeal, carotid, prevertebral or pretracheal space and further into the mediastinum [2, 3]. Although many deep neck infections are prevented by the widespread use of antibiotics on the initial infections today, it is still a severe and progressive infection with, especially when descending into the mediastinum, high mortality rates of 10–40% due to sepsis and organ failure [4, 5]. Because of the oral and pharyngeal origin of these infections, the most common pathogens responsible are aerobic bacteria like *Streptococcus pyogenes* and *Staphylococcus aureus*, while anaerobic bacteria are found less frequently [6–8]. Several risk factors for the development of severe deep neck infections and an affection of the mediastinum have been identified, such as age older than 55 years [9], cardiopulmonary comorbidities [10], nutritional status [11] or especially diabetes mellitus [12]. While the original infections can be diagnosed quickly due to the specific symptoms, the diagnosis of the spreading along the cervical fascial planes into the mediastinum is often delayed because of the unspecific additional symptoms until clinical deterioration [3, 4]. Therefore, computed tomography is essential for confirmation of abscess formations in the deep neck or the mediastinum [13].

There is a broad consensus regarding the need for multidisciplinary treatment of severe deep neck infections. Empiric antibiotic treatment should begin before definitive microbiological results are available, and should cover Gram-positive as well as Gram-negative bacteria [14]. While some studies suggest starting only with antibiotic therapy [15, 16], most authors agree on the need for surgical drainage of the abscess formations, which, besides its immense therapeutic significance, also provides microbiological samples to better suit antibiotic therapy to the causative microorganisms [2, 3, 17]. Several questions regarding the surgical therapy are still under debate, for example the interval between hospitalization and first intervention [17], the frequency of surgical debridement [18], or the need for regular lavage of the drained fascial spaces [2, 10, 19]. For mediastinal involvement, the surgical route is also object of discussion, with some authors favoring trans-cervical [10, 18] and others recommending a transthoracic approach [2, 19]. While some attempts at creating an algorithm have been done [4, 17], to the best of our knowledge, they have not been widely accepted. This may be attributed to the rarity and the variable presentation of the disease, as well as the different specialities (head and neck surgery, thoracic surgery, internal medicine and infectiology) involved in the treatment in different hospitals.

The aim of the present study was to analyze the management and outcome of 218 patients with deep neck infections treated with cervical surgery alone at our institution retrospectively and to propose an algorithm for diagnostic and therapy according to the results.

## Methods

After obtaining approval of the Würzburg University´s Hospital Institutional Review Board and local ethics committee, the charts of all patients treated at our institution with an abscess of the deep neck between January 2009 and December 2019 were reviewed. Patients without a spread through the cervical fascial spaces, like isolated peritonsillar or prevertebral abscesses, were excluded. Altogether, 218 patients could be identified who matched the inclusion criteria.

Baseline patient characteristics investigated included age, gender, medical history, body mass index (BMI) as well as duration of symptoms and therapy prior to hospitalization. Among the laboratory parameters, especially leukocytes, C-reactive protein (CRP) and procalcitonin (PCT) were evaluated. All patients were treated with systemic intravenous antibiotics consisting either of ampicillin and sulbactam or cefazolin and metronidazole, and all underwent surgery on the day of admission to the hospital. Surgery was performed either by ENT surgeons alone or, in addition, by thoracic surgeons and consisted of the drainage of all abscess formations including the mediastinum via a collar approach or via thoracotomy. If applicable, enoral drainage of the focus of infection via tonsillectomy or endoscopic incision of the parapharyngeal space was also conducted. Drainage tubes were placed in all cervical abscess locations, and afterwards were rinsed with antiseptic fluids and sodium chloride 3 times a day. After removal of the drainage tubes, the resulting hole in the neck wound was left to secondary wound closure rather than surgical closure, if possible. Besides the antibiotics administered, the need for a change of substance according to resistances as well as the overall duration of antibiotic therapy was analyzed. Bacterial results in the initial sampling and, if applicable, in the clinical course in case of deterioration, were reviewed. Frequency and extent of surgical intervention, duration and frequency of lavages through drainage tubes, complications of surgery, airway management and need for tracheotomy were investigated. Regarding the clinical course, we analyzed the duration of hospitalization, prolonged morbidity after reconvalescence from the infection like a persisting tracheotomy or feeding tube, and the mortality rate. Patients with a mediastinal involvement were analyzed separately and were compared to those without.

Data collected were transferred to standard spreadsheets and statistically analyzed using GraphPad prism software (version 6.0e, GraphPad Software Inc., San Diego, CA, USA). DÁgostino-Pearson omnibus normality test was conducted to verify normality distribution. Due to the normal distribution, the Student’s *t* test was applied when comparing two groups with continuous variables. Categorical data were analyzed with Fisher’s exact test, or Chi-squared test for more than two variables. For odds ratio analysis, the continuous variables of age, BMI, initial CRP and initial PCT were converted into binaries. A value of *p* < 0.05 was considered statistically significant. When testing multiple comparisons, a Bonferroni correction was applied to each group of tests.

## Results

A total of 218 patients were analyzed matching the inclusion criteria. 173 patients (79.36%) had deep neck infections restricted to abscesses in the cervical spaces, while 45 patients (20.64%) had abscess formations in the mediastinum. Table 1 shows patient characteristics and initial findings in patients with and without mediastinal involvement. Mean age was 55.25 years vs. 61.33 years (*p* = 0.048, not significant after Bonferroni correction), mean BMI was 26.86 vs. 28.16 kg/m^2^ (*p* = 0.173). Male to female ratio was 49.71/50.29% vs. 60.00/40.00% (*p* = 0.244). 41.86% of the patients without mediastinal involvement hat a medical history compared to 60.00% in the group with mediastinal involvement (*p* = 0.043, not significant after Bonferroni correction). A focus of infection could be identified in 72.09% vs 100% of the cases (*p* < 0.001). In patients with mediastinal involvement, the most frequently found focal infections were the tonsils (55.56%) and the teeth (15.56%). In patients with only cervical abscesses, the salivary glands played the most important role (19.19%) and the percentage of dental foci was the same (14.53%), while tonsils were less frequently identified as a focus (7.56%). Initial blood sampling revealed leukocytes of 13.450/µl vs. 17.390/µl (*p* = 0.105), CRP of 10.41 mg/dl vs. 26.09 mg/dl (*p* < 0.001) and PCT of 0.33 ng/ml vs. 10.76 ng/ml (*p* < 0.001).

**Table 1:** Baseline patient characteristics and initial clinical findings in patients with and without mediastinal involvement of a deep neck infection

	No mediastinal involvement	Mediastinal involvement	*p*-value
Total patients	173 (79.36%)	45 (20.64%)	
Gender			0.244
Male	86 (49.71%)	27 (60.00%)	
Female	87 (50.29%)	18 (40.00%)	
Age in years	55.25	61.33	0.048
BMI in kg/m^2^	26.86	28.16	0.173
Medical history	72 (41.86%)	27 (60.00%)	0.043
Diabetes mellitus	21 (12.21%)	12 (26.67%)	
Cancer	20 (11.63%)	7 (15.56%)	
Other immunosuppressive disease	11 (6.40%)	7 (15.56%)	
Focus			* < 0.001
Tonsils	13 (7.56%)	25 (55.56%)	
Teeth	25 (14.53%)	7 (15.56%)	
Salivary gland	33 (19.19%)	0	
Other	53 (30.81%)	13 (28.89%)	
No focus identified	48 (27.91%)	0	
Initial inflammation values			
Leukocytes in 1000/µl	13.45	17.39	0.105
CRP in mg/dl	10.41	26.09	* < 0.001
PCT in ng/ml	0.33	10.76	* < 0.001

Values are given in number (proportion) or mean

*BMI* body mass index, *CRP* C-reactive protein, *PCT* procalcitonin

*Indicates significance after Bonferroni correction

**Fig. 1:**
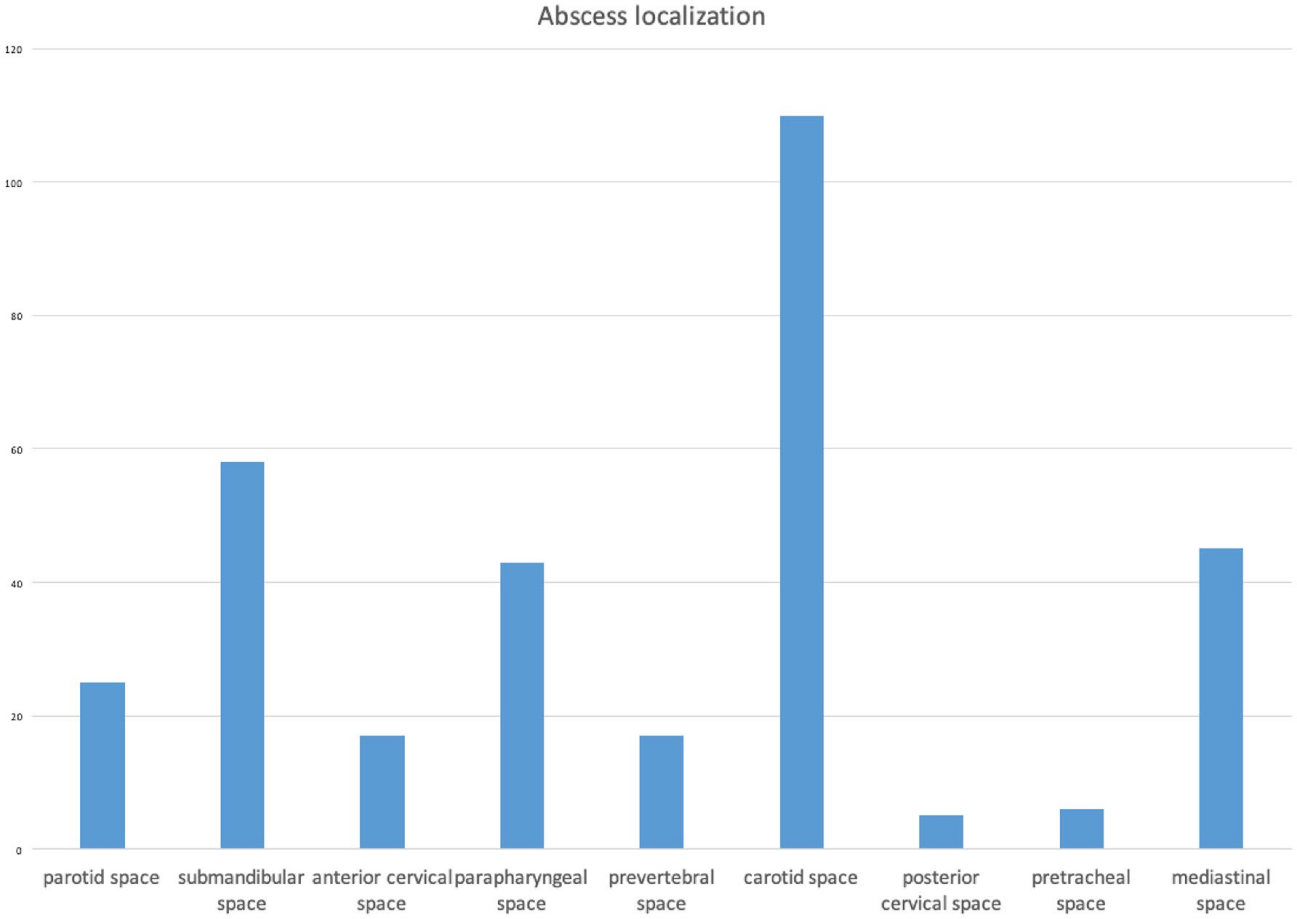
Distribution of the abscess localizations. Because of multi-space involvement, the total number exceeds the 218 patients

Figure 1 depicts the distribution of the abscess localizations in the patients analyzed. Most abscess formations were found in the carotid space (110 patients, 50.46%), followed by the submandibular space (58 patients, 26.61%), mediastinal space (45 patients, 20.64%), parapharyngeal space (43 patients, 19.72%) and parotid space (25 patients, 11.47%). Bilateral abscesses were present in 52 patients (23.85%). As expected, mediastinal involvement never occurred without an additional abscess formation in other cervical spaces.

The results of bacterial sampling are shown in Table 2. In patients with mediastinal involvement, polymicrobial infections could be identified in 40% of the cases vs. only 5.8% in patients without mediastinal involvement. Although predominantly *Streptococcus viridans* and ß-hemolytic streptococci were found in mediastinal abscess samples, the variety of microorganisms (8.9% each for *staphylococcus aureus*, Coagulase-negative staphylococcus, *Prevotella* spec. and Enterobacteriaceae, respectively, as well as 31.1% other bacteria) was higher than in patients without mediastinal abscess formation. Also, the rate of multidrug-resistant organisms was higher in these patients (8.9% vs. 1.2%). No bacterial growth from the samples occurred in 39.9% of patients with only cervical abscesses and in 22.2% of patients with mediastinal involvement.

**Table 2:** Microbacterial results in patients with and without mediastinal involvement of deep neck infections

	No mediastinal involvement		mediastinal involvement	
*Streptococcus viridans*	37	21.2%	18	40%
*Staphylococcus aureus*	25	14.1%	4	8.9%
ß-hemolytic streptococci	13	7.5%	9	20%
Coagulase-negative staphylococcus	9	5.2%	4	8.9%
*Haemophilus* species	5	2.9%	2	4.4%
*Prevotella* species	3	1.7%	4	8.9%
*Fusobacterium* species	4	2.3%	2	4.4%
*Peptostreptococcus*	4	2.3%	0	0
Enterobacteriaceae	0	0	4	8.9%
Other	15	8.7%	14	31.1%
Multidrug-resistant organisms (MDRO)	2	1.2%	4	8.9%
No bacterial growth in culture	69	39.9%	10	22.2%
Polymicrobial	10	5.8%	18	40%

Values are given in numbers and proportion. Proportions can exceed 100% due to polymicrobial infections

The diagnostic measures, therapy and clinical course of the patients involved is presented in Table 3. Patients with mediastinal abscesses had significantly more CT-scans (3.58 vs. 0.85, *p* < 0.001), a longer hospitalization (39.78 vs. 9.79 days, *p* < 0.001) and a longer duration of antibiotic therapy (30.11 vs. 8.98 days, *p* < 0.001). In patients with mediastinal involvement, the empiric antibiotic therapy was suitable for the pathogen responsible in 73.33% compared to 93.06% (*p* < 0.001, OR 5.32 [2.16–13.09]). Antibiotic regimen was changed over the course of therapy in 44.44% of the mediastinal cases compared to 6.36% (*p* < 0.001, OR 11.71 [5.01–27.34]). As expected, mediastinal abscesses required more surgical interventions (4.27 vs. 1.11, *p* < 0.001), a prolonged wound lavage (11.78 vs. 4.06 days, *p* < 0.001) and a higher rate of tracheostomies (82.22% vs. 3.47%, OR 128.7 [42.12–393.4]). Five of 45 patients with mediastinal abscesses needed a transthoracic approach by a thoracic surgeon (11.11%). A necrotizing fasciitis was only found in patients with mediastinal involvement, and there in nine of 45 patients (20.00% vs. 0%, *p* < 0.001). Consecutively, the four fatal cases in this collective were all patients with mediastinal involvement and a necrotizing fasciitis who needed transthoracic surgery, while there were no casualties in the group with only cervical abscesses (*p* < 0.002, OR 37.63 [1.99–713.2]). Initial airway management consisted in only seven cases of tracheotomy in local anesthesia (3.21%) and six cases of fiberoptic transnasal intubation (2.75%), while the rest of the patients could have a conventional or video-laryngoscopic oral intubation (94.04%).

**Table 3:** Diagnostic, therapy and clinical course of patients with or without mediastinal involvement in deep neck infections

	No mediastinal involvement	Mediastinal involvement	*p*-value	OR (95% CI)
Number of CT-scans	0.85	3.58	* < 0.001	
Hospitalization in days	9.79	39.78	* < 0.001	
Antibiotic therapy in days	8.98	30.11	* < 0.001	
Empiric therapy suitable for pathogen	161 (93.06%)	33 (73.33%)	* < 0.001	5.32 (2.16–13.09)
Need for a change of antibiotic regimen	11 (6.36%)	20 (44.44%)	* < 0.001	11.71 (5.01–27.34)
Number of surgeries	1.11	4.27	* < 0.001	
Need for transthoracic surgery	0	5 (11.11%)		
Wound lavage in days	4.06	11.78	* < 0.001	
Mortality	0	4 (8.89%)	*0.002	37.63 (1.99–713.2)
Necrotizing fasciitis	0	9 (20.00%)	* < 0.001	90.32 (5.14–1588)
Tracheotomy	6 (3.47%)	37 (82.22%)	* < 0.001	128.7 (42.12–393.4)

Values are given in numbers (proportion) or mean. For categorical data, odds ratio with the respective 95% confidence interval have been calculated

*Indicates significance after Bonferroni correction

Finally, risk factors for severe clinical courses were analyzed (Table 4). Parameters chosen to indicate a severe clinical course were hospitalization longer than 10 days, more than one surgery needed as well as the need for a tracheostomy. Longer hospitalization was correlated with mediastinal involvement (*p* < 0.001, OR 32.44 [10.95–96.10], initial CRP (*p* < 0.001, OR 6.34 [3.41–11.79]), initial PCT (*p* < 0.001, OR 21.71 [7.30–64.59]) and a medical history (*p* < 0.001, OR 2.78 [1.58–4.90]). The number of surgeries was correlated with mediastinal involvement (*p* < 0.001, OR 137.5 [42.14–448.7]), as well as initial CRP (*p* < 0.001, OR 4.88 [2.38–9.99]) and PCT (*p* < 0.001, OR 11.20 [5.20–24.15]). The need for a tracheostomy was positively correlated with age higher than 60 years (*p* = 0.007, OR 2.61 [1.30–5.23]), mediastinal involvement (*p* < 0.001, OR 128.7 [42.12–393.4]), initial CRP (*p* < 0.001, OR 11.68 [4.38–31.13]), initial PCT (*p* < 0.001, OR 25.24 [10.42–61.09]) and a medical history (*p* = 0.004, OR 2.83 [1.40–5.72]). All these aforementioned significances also passed the Bonferroni correction. BMI did not show any significant correlation towards these parameters. Only mediastinal involvement, initial CRP and initial PCT were positively correlated to all three parameters.

**Table 4:** Risk factors for long hospitalization, more surgeries and tracheotomy for all patients

	Long hospitalization		Number of surgeries		Tracheotomy	
	*p*-value	OR (95% CI)	*p*-value	OR (95% CI)	*p*-value	OR (95% CI)
Age > 60 years	0.048	1.79 (1.03–3.13)	0.205	1.56 (0.84–2.91)	*0.007	2.61 (1.30–5.23)
BMI > 30 kg/m^2^	0.055	1.86 (1.00–3.45)	0.357	1.42 (0.70–2.87)	0.705	1.15 (0.55–2.41)
Mediastinal involvement	* < 0.001	32.44 (10.95–96.10)	* < 0.001	137.5 (42.14–448.7)	* < 0.001	128.7 (42.12–393.4)
Initial CRP > 10.00 mg/dl	* < 0.001	6.34 (3.41–11.79)	* < 0.001	4.88 (2.38–9.99)	* < 0.001	11.68 (4.38–31.13)
Initial PCT > 0.50 ng/ml	* < 0.001	21.71 (7.30–64.59)	* < 0.001	11.20 (5.20–24.15)	* < 0.001	25.24 (10.42–61.09)
Medical history	* < 0.001	2.78 (1.58–4.90)	0.017	2.24 (1.186–4.23)	*0.004	2.83 (1.40–5.72)

For OR-analysis, the items age, BMI, initial CRP and initial PCT have been converted to binaries by defining cut-off values

*BMI* body mass index, *CRP* C-reactive protein, *PCT* procalcitonin

*Indicates significance after Bonferroni correction

**Fig. 2:**
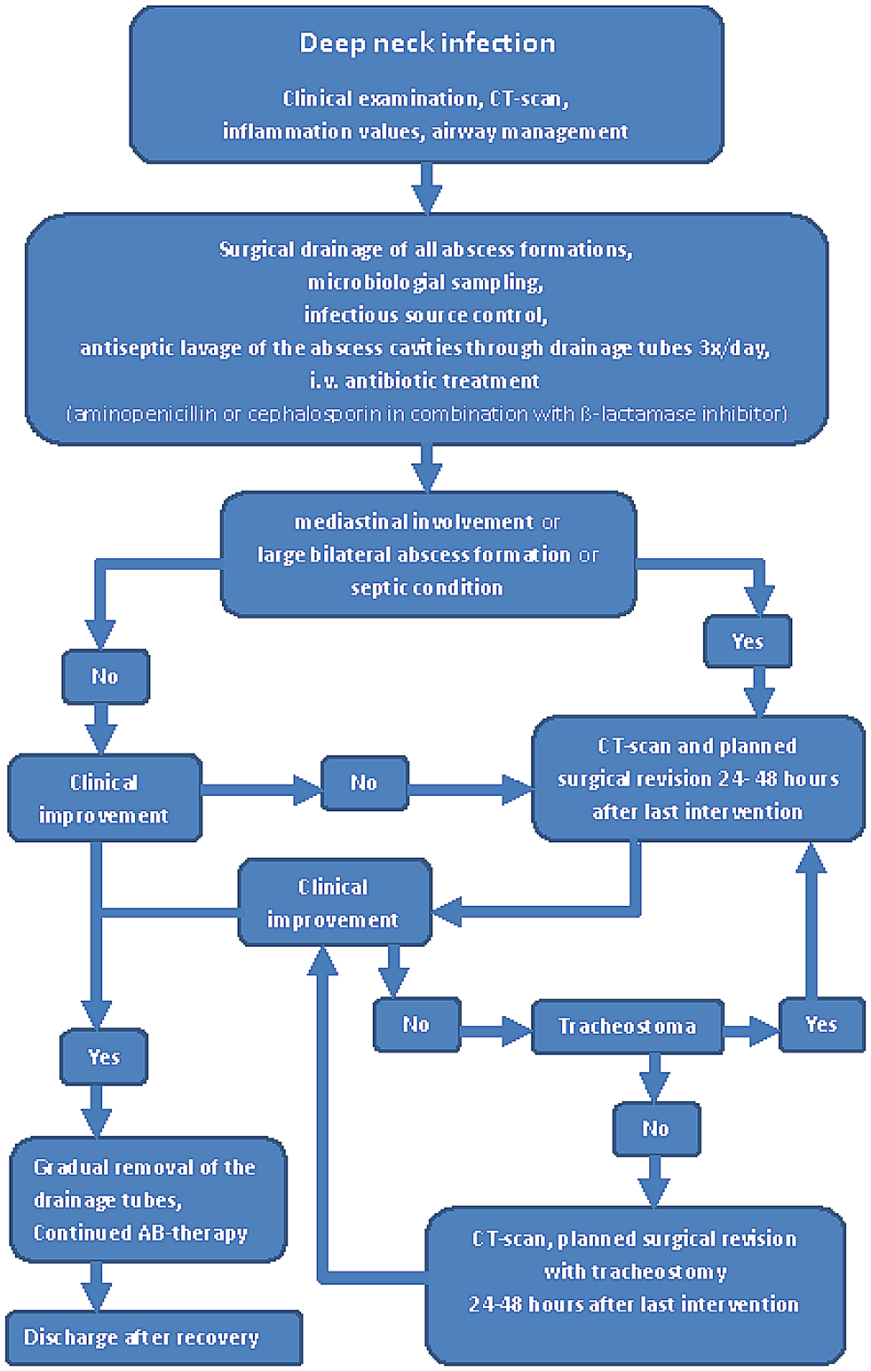
Proposed algorithm for diagnostic and treatment of cervical abscesses

Regarding long-term complications, decannulation of patients with tracheostomy during the hospitalization was possible in 33 of 43 patients (73.33%). nine patients still needed a feeding tube at time of discharge because of swallowing disorders (4.13%). Surgical closure after wound healing disorders was necessary in seven patients (3.21%) and vacuum-assisted therapy in four patients (1.83%). One patient needed extended surgery with a major pectoral flap and vascular reconstruction due to severe bleeding following an infectious arrosion of the common carotid artery. According to these results, we created an algorithm to reflect our recommended diagnostic and therapeutic pathways (Fig. 2).

## Discussion

Deep neck infections are still recognized as potentially life-threatening diseases. While in the era of antibiotics many deep neck infections are prevented by treating the initial infection early on, the increased aging in the populations worldwide has led to a certain increase in the incidence of severe manifestations, mainly due to comorbidities [9]. Thus, a standardized diagnostic and therapeutic approach for these patients is warranted.

Most of these infections have their origin at an infectious focus. The main etiology in the present study were teeth, tonsils and salivary glands, with a higher percentage of tonsillar infections in patients with mediastinal involvement. While several authors reported on predominantly dental infections, especially in severe cases with mediastinal infections or necrotizing fasciitis [1, 9, 20], others demonstrate higher percentages of tonsillar [15, 21] or salivary [22] origins of infection, or a connection to injection drug use [23]. This variation in the reported pathogeneses suggest regional or demographic differences [12, 18, 23, 24], or may be caused by patient selection due to the different specialities involved [16, 25, 26]. According to the literature, in 17–67% of all patients no focus of infection could be identified [21, 22, 25], which is in accordance to the present study’s 27.91% and may be related to prior outpatient treatment of the original infection [12]; however, in our patients with mediastinal involvement, a focus of infection could be found in every patient.

The pattern of spreading through the fascial spaces is connected to the origin of infection; dental infections tend to spread through the submandibular space, tonsillar infections often follow the parapharyngeal and carotid spaces and salivary abscesses expand through parotid, submandibular and carotid spaces [1, 27]. Accordingly, abscess formation in the present study was mainly localized in carotid, submandibular, parapharyngeal and parotid spaces. Involvement of the mediastinum was shown in 20.64%, which is somewhat higher than the proportions reported in literature with 4–11% [11, 14]. Besides regional or demographic differences, this might also be a selection bias, since our institution as a tertiary university hospital probably has a higher proportion of severe cases.

Since cervical abscesses originate from infections of teeth, tonsils or salivary glands, the bacterial spectrum is largely coherent with the microorganisms found in these specified locations. *Streptococcus viridans*, *staphylococcus aureus* and ß-hemolytic streptococci make up for the majority of microorganisms causative for deep neck infections in the present study as well as in most of the literature [14, 15]. Likewise, in severe cases with mediastinal involvement or necrotizing fasciitis, higher percentages of gram-negative and/or anaerobic bacteria as well as more polymicrobial infections have been described [9, 12, 21], which could also be shown in the present study. The relatively high proportion of patients with no bacterial growth in culture is also in accordance with the majority of studies regarding this topic [1, 9, 14] and might also be related to prior antibiotic outpatient treatment.

While there is a broad consensus regarding the need for multidisciplinary treatment of deep neck infections, several questions about the therapy involved are still under debate. At our institution, and therefore in the present study, all patients with radiologically proven cervical or mediastinal abscess formation receives surgery on the day of admission. Several authors discuss the possibility of first starting with antibiotic therapy alone, and only recommend surgery if the patients show signs of clinical deterioration [16, 28]. Others regard a needle aspiration of the abscesses to be sufficient in some cases [1, 29]. In these studies, however, often no clear difference between deep neck infections presenting as a cellulitis alone or with already existing abscess formations have been made, possibly explaining the success rates with antibiotics alone [1, 16, 28]. In the present study, all patients had confirmed abscess formations. The majority of studies favor surgical drainage of the abscesses on the day of admission [14, 30–32], and even report on increased morbidity and mortality connected to delayed surgical therapy [15, 30]. Planned surgical revisions with drainage are also thought to be an integral part of the therapy, especially in severe cases [4, 14, 15, 20, 30]. Regular lavage with antiseptic fluids, as was done in the present study, is also recommended by several authors [14, 15], as is a negative pressure drainage in some cases [15].

In the algorithm presented here, we put a strong emphasis on an early tracheostomy to facilitate surgical revisions and reduce sedation time for the patients. Though Barber et al. report on a correlation of tracheostomy with longer hospitalization [33], this is mainly attributed to the severity of the cases when needing a tracheostomy. On the other hand, patients with an early tracheostomy showed a shorter duration of ICU care and overall morbidity and mortality in several studies [20, 34, 35].

Several risk factors for a severe clinical course have been identified, for example age older than 55 years [4, 9], cardiopulmonary disease [10], nutritional status [11] or diabetes mellitus [4, 12]. In the present study, age and medical history (among which were many cardiopulmonary diseases and of course diabetes mellitus) only partially correlated with severity of the disease. The factors best predicting a severe clinical course were mediastinal involvement of the abscesses and the initial CRP and PCT values. Mediastinal abscess formation has been shown as an independent risk factor by several authors [4, 11, 15, 36], while a higher initial CRP was also correlated to severe clinical courses [21]. PCT has not been described in this regard, possibly because it is not regularly analyzed for all patients with cervical abscesses in many institutions.

One advantage of the present study is the large number of patients included, the standardized patient management and the completeness of data. A limiting factor is its retrospective design, and that there was no matching of the groups, both of which may be explained by the emergency nature of the disease and the variable clinical presentation. Nevertheless, in our opinion it gives a thorough overview of this severe disease as well as its diagnostic, therapy and outcome.

In conclusion, the present study showed in a large retrospective analysis that patients with mediastinal involvement in deep neck infections show distinct differences regarding the infectious focus, microbiological results, initial inflammation values, extent of surgical therapy needed and outcomes like tracheostomy and mortality compared to patients with only cervical manifestations. Mediastinal involvement as well as initial CRP and PCT are good predictors for severe clinical courses. We emphasize immediate surgical drainage, broad empiric antibiotic therapy, regular planned surgical revisions, antiseptic wound lavage and an early tracheostomy as the cornerstones of therapy in these patients. The presented algorithm can be a valuable tool in standardizing diagnostic and treatment of this severe disease.

## Data Availability

The datasets used and/or analyzed during the current study are available from the corresponding author on reasonable request.
